# Hydrogen sulfide in respiratory health: therapeutic insights from sulfur-rich thermal waters

**DOI:** 10.4103/mgr.MEDGASRES-D-25-00119

**Published:** 2026-03-24

**Authors:** Michele Antonelli, Davide Donelli

**Affiliations:** Studio Medico Antonelli, Cavriago, RE, Italy; World Hydrothermal Organisation (OMTh), Levico Terme, TN, Italy; Studio Cardiologico Donelli, Cavriago, RE, Italy

Hydrogen sulfide (H_2_S), traditionally regarded as a toxic gas, is now recognized as a crucial gasotransmitter with concentration-dependent effects on health. In the respiratory system, H_2_S homeostasis is particularly important: low levels support antioxidant defenses, mitochondrial dynamics, immune functions, and epithelial barrier integrity, while excessive accumulation can drive cytotoxicity and inflammation. Disruptions in this balance, whether from endogenous dysregulation or altered microbial production, may be implicated in several chronic disorders. Therapeutically, sulfur-rich thermal waters (STWs) provide an exogenous source of H_2_S that has long been applied in respiratory care. Clinical studies support STW aerosol inhalation as a safe, integrative and non-pharmacological intervention capable of modulating inflammatory pathways, enhancing mucociliary clearance, and improving outcomes in conditions such as allergic rhinitis, rhinosinusitis, and chronic obstructive pulmonary disease. Integrating STW-based balneotherapy within the framework of H_2_S homeostasis highlights its dual role as a modulator of cellular physiology and a potential therapeutic intervention, underscoring the need for more studies to better define optimal dosing and long-term benefits.

The role of gasotransmitters in mammalian physiology has garnered increasing attention over the last few decades, particularly following the recognition of nitric oxide and carbon monoxide as key signaling molecules. The more recently acknowledged H_2_S, once primarily regarded as a toxic environmental pollutant, has now emerged as a vital biological regulator, transforming our understanding of cellular signaling, redox homeostasis, and inflammatory modulation. Intriguingly, H_2_S was abundantly present in the primordial Earth’s atmosphere, and the earliest forms of life not only endured but thrived in environments rich in reactive gases such as nitric oxide, carbon monoxide, and H_2_S: over evolutionary time, organisms have developed the ability to harness these gases as integral components of physiological regulation. These ancient adaptations have been refined and conserved throughout evolution, with H_2_S now known to play extensive systemic roles in mammals, ranging from respiratory functions to neurotransmission, metabolic control, cardiovascular regulation, and immune system modulation. In the present work, however, we will focus specifically on its role in the respiratory system.

H_2_S is endogenously synthesized through both enzymatic and non-enzymatic pathways. The principal enzymes involved in its production are cystathionine β-synthase, cystathionine γ-lyase, and 3-mercaptopyruvate sulfurtransferase, frequently acting in conjunction with cysteine aminotransferase.^1^ These enzymes localize in various cellular compartments, including the cytoplasm and mitochondria, and utilize substrates such as L-cysteine and D-cysteine. In particular, D-cysteine is metabolized through the action of D-amino acid oxidase, a lesser-known but significant route of H_2_S generation. Although non-enzymatic mechanisms of H_2_S synthesis are quantitatively minor, they become increasingly relevant under conditions of oxidative stress or altered redox balance. In addition to host-derived sources, H_2_S is also produced by gut microbiota through the reduction of sulfur-containing dietary compounds.^2^ This microbial source of H_2_S contributes importantly to systemic homeostasis, particularly in the regulation of cardiometabolic functions. Disruptions in gut microbial populations, commonly referred to as dysbiosis, have been shown to significantly alter H_2_S bioavailability and may be potentially associated with inflammatory, metabolic, degenerative, and even proliferative conditions.^3^ H_2_S can be generated by sulfate-reducing bacteria of the gut microbiota such as *Desulfovibrio*^3^: these microorganisms synthesize H_2_S via dissimilatory sulfate reduction, and their activity can be influenced by dietary intake of sulfur-containing compounds, including amino acids like cysteine and methionine, as well as sulfated polysaccharides found in certain vegetables and processed foods. Dietary composition strongly influences intestinal H_2_S production, with fiber-rich, plant-based proteins generally suppressing H_2_S levels, while low-fiber, animal-based proteins and highly processed Western-style diets tend to excessively elevate them.^2^ At physiological levels, microbiota-derived H_2_S contributes to systemic homeostasis by supporting epithelial barrier integrity, regulating immune responses, and maintaining redox balance. Excessive H_2_S production under dysbiosis or unbalanced diet may promote low-grade systemic inflammation, oxidative stress, and immune dysregulation, potentially exacerbating inflammatory disorders.

The pleiotropic physiological actions of H_2_S are particularly notable in the respiratory system. Inflammatory airway diseases such as allergic rhinitis, chronic rhinosinusitis, bronchial asthma, and chronic obstructive pulmonary disease (COPD) all exhibit measurable alterations in H_2_S metabolism: in allergic rhinitis, for instance, elevated levels of both cystathionine β-synthase and cystathionine γ-lyase have been detected in the nasal mucosa, correlating with increased local concentrations of H_2_S.^4^ This upregulation appears to be a compensatory response aimed at mitigating the chronic inflammatory environment characteristic of allergic rhinitis. Histologically, cystathionine β-synthase is primarily located in the superficial nasal epithelium and submucosal glands, while cystathionine γ-lyase is confined to the vascular endothelium and adjacent smooth muscle cells. This anatomical distribution supports a complex role for H_2_S in both immune defense and vascular homeostasis within the nasal passages. Although clinical data remain limited, preliminary studies suggest that treatment with STWs, administered as aerosol inhalations or balneotherapy, can significantly alleviate symptoms in patients with allergic rhinitis and rhinosinusitis.^5^,^6^ These treatments have been associated with decreased serum IgE levels and increased IgA levels, suggesting an immunomodulatory mechanism that shifts the immune response from an allergen-driven Th2 phenotype to a more regulated mucosal immune profile.^7^ Improvements in nasal airflow resistance and mucociliary transport times further underscore the potential for STWs to augment the mucosal barrier and reduce inflammatory load. For example, a recently published case series reports on eight adult patients with rhinogenic deafness caused by chronic rhinosinusitis who underwent a 2-week treatment cycle at “Terme di Frasassi - S. Vittore” in Italy during the 2024 season.^6^ The intervention consisted of daily STW aerosols and bilateral tubaric insufflations. All patients initially presented with tympanometric curves of type B or C, consistent with Eustachian tube dysfunction. Post-treatment evaluation demonstrated normalization to type A curves in all cases, indicating restored middle ear ventilation. Patients reported subjective improvement in ear fullness, with a median improvement score of 9.5 out of 10. Benefits were also observed in individuals with comorbid chronic metabolic or inflammatory disorders, and no adverse effects were documented. Similarly, in COPD, systemic and localized H_2_S levels have been found to fluctuate depending on disease activity.^8^ Stable COPD patients tend to have higher baseline levels of H_2_S compared to those experiencing exacerbations, particularly those triggered by bacterial or viral infections. This suggests that endogenous H_2_S production is dynamically regulated in response to inflammatory stimuli and may function as an intrinsic defense mechanism. During acute disease phases, however, the biosynthetic capacity of H_2_S-producing enzymes may be insufficient or impaired. Therapeutic interventions using STWs in COPD patients have led to notable reductions in oxidative stress biomarkers, along with subjective and objective improvements in respiratory function.^4^ These effects are particularly salient in moderate to severe disease states, where conventional pharmacological interventions often become less effective or induce undesirable side effects. A cohort study involving current and former heavy smokers further highlights the therapeutic potential of sulfurous inhalation therapy.^9^ After a 10-day course of STW aerosol inhalation, biochemical analysis of exhaled breath condensate revealed a significant metabolic shift: increased citrulline and decreased ornithine levels, indicating reduced arginine-to-nitric oxide conversion and an overall anti-inflammatory metabolic profile. Despite the absence of immediate changes in spirometric indices, these molecular shifts suggest early-stage improvement in the inflammatory milieu and cellular metabolic function, possibly reflecting enhanced epithelial resilience and repair capacity.

The biological actions of H_2_S, while systemic, have particular relevance for the respiratory system: its ability to regulate oxidative stress and mitochondrial dynamics supports epithelial cell integrity and adaptation to inflammatory or allergen-induced stress in the airways. The concentration-dependent nature of H_2_S is especially important in the respiratory tract: at low levels it promotes antioxidant defenses and energy efficiency, whereas at higher levels it can contribute to cytotoxicity. Historically perceived as a toxic gas, H_2_S is now being reassessed in light of emerging data supporting its therapeutic potential. In balneotherapy, STWs have long been utilized for their mucolytic and anti-inflammatory properties.^10^ Modern investigations are beginning to validate these traditional practices with scientific rigor. For individuals with chronic inflammatory respiratory conditions who fail to achieve optimal control with pharmacological regimens, adjunctive therapy with STWs may offer a safe and effective option. Furthermore, unlike systemic corticosteroids and long-term antibiotic use, STWs are generally well-tolerated and devoid of severe systemic side effects. Importantly, the therapeutic context in which STW treatments are administered should not be overlooked. Health resorts often offer psychological and behavioral benefits that contribute to overall well-being.^10^ The combination of structured treatment protocols, physical relaxation, reduced environmental stressors, and improved health awareness can synergistically enhance the physiological benefits of H_2_S exposure. These holistic elements, while difficult to quantify, are nonetheless integral to the observed clinical improvements in many patients undergoing balneotherapy.

**Figure 1:**
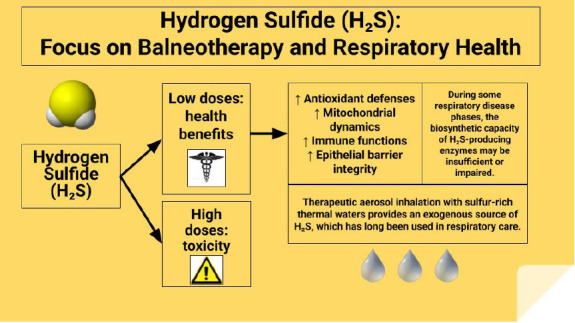
Hydrogen sulfide and its biological significance. Created in Google Slides with exclusively Public Domain images.

In conclusion, H_2_S is a molecule of profound biological significance, bridging the ancient biochemical landscapes of early Earth with the sophisticated signaling networks of contemporary human physiology (**Figure 1**). Its endogenous production, microbial interactions, and therapeutic applications span multiple domains of health and disease. Recent research has underscored the necessity of H_2_S homeostasis, since both excess and deficiency can be detrimental (too much H_2_S disrupts cellular function and promotes inflammation, while too little deprives tissues of its protective, signaling, and metabolic roles). As our understanding of its mechanisms continues to evolve, so too does its promise as a cornerstone in novel therapeutic approaches. Harnessing the full potential of H_2_S will require a multidisciplinary effort, integrating molecular biology, clinical research, environmental science, and traditional medicine. Future studies should aim to elucidate the specific cellular pathways influenced by H_2_S in various disease contexts, explore its interactions with other gasotransmitters, and define standardized treatment protocols that maximize therapeutic efficacy while minimizing risks. Ultimately, H_2_S may redefine our integrative approach to managing a wide array of inflammatory and degenerative conditions.

*Michele Antonelli served as external scientific consultant for “Terme di Frasassi - S. Vittore”, (AN, Italy). The Direction of “Terme di Frasassi” had no role in the collection, management, or publication of data related to this work.*
